# Efficacy Assessment of Ultrasound Guided Lauromacrogol Injection for Ablation of Benign Cystic and Predominantly Cystic Thyroid Nodules

**DOI:** 10.3389/fphar.2019.00478

**Published:** 2019-05-08

**Authors:** Yijie Dong, Jianqiao Zhou, Zhenhua Liu, Ting Luo, Weiwei Zhan

**Affiliations:** Ruijin Hospital, School of Medicine, Shanghai Jiao Tong University, Shanghai, China

**Keywords:** ultrasound, lauromacrogol, ablation, thyroid, nodules

## Abstract

**Background:**

To assess the efficacy and safeness of ultrasound guided lauromacrogol injection for ablation of benign cystic and predominantly cystic thyroid nodules.

**Methods:**

From July 2016 to July 2018, 102 patients with 107 nodules were treated with ultrasound guided lauromacrogol injections for ablation and 43 nodules completed at least 12 months follow-up. Nodules sonographic characteristics, volume changes before and after USG-LIA, and complications were evaluated.

**Results:**

Mean nodule volume decreased from 17.27 ± 20.51 ml to 5.35 ± 14.68ml (*P* < 0.05), and the overall resolution rate (volume reduction rate > 50%) was 91.67% in purely cysts and 75.90% in predominantly cystic nodules at the last follow-up. Within 6 months after treatment, the volume of the target nodule at each follow-up was smaller than the previous one (*P* < 0.001 for all). However, there was no significant difference of volume change between the 6th month and the 12th month. No severe complications occurred in this study.

**Conclusion:**

Ultrasound guided lauromacrogol injection for ablation is an effective and safe treatment modality in both purely cystic and predominantly cystic thyroid nodules.

## Introduction

Cystic nodules are very common in benign thyroid nodules, which could cause symptomatic and cosmetic problems in 60–90% patients (Bennedbaek and Hegedus, 2003; Valcavi and Frasoldati, 2004). Surgery is the ultimate treatment for intractable benign cystic nodules. In addition to surgery, simple fine-needle aspiration, saline injection and ultrasound guided percutaneous ethanol injection (USG-PEI) are other alternative treatment options. It was reported that simple aspiration and saline injection had only 7–38% success rate. As a comparison, mainly USG-PEI had 75–86.3% success rate (Valcavi and Frasoldati, 2004; Sung et al., 2013; Reverter et al., 2015). However, USG-PEI had a high recurrence rate in predominantly cystic nodules and complications included flushing, dizziness, and dysphonia, and even several ethyl toxic necrosis caused by ethanol leakage to surrounding tissues (Mauz et al., 2005; Jang et al., 2012; Suh et al., 2015). Lauromacrogol, another type of chemical sclerosants different from alcohol, has been widely used in esophageal variceal bleeding, varicose vein. Literature has also reported lauromacrogol treatments in cystic lesion of viscera such as hepatic cysts, renal cysts and pancreatic cystic neoplasms (Moser et al., 2013; Xue and Geng, 2015; Yonguc et al., 2015; Linghu et al., 2017; Star et al., 2018). However, lauromacrogol was rarely reported in the treatment of thyroid nodules.

Therefore, the aim of this study was to evaluate the efficacy and safeness of ultrasound guided lauromacrogol injection for ablation (USG-LIA) of cystic and predominantly cystic thyroid nodules.

## Materials and Methods

### Patients

This prospective study was approved by our Institutional Review Board. From July 2016 to July 2018, 117 patients with symptomatic or aesthetical nodules which ultrasound confirmed purely cystic and predominantly cystic thyroid nodules were recommended for USG-LIA. Nodules with solid components less than 10% were defined as purely cyst. Nodules with solid components 10–50% were defined as predominantly cyst (Gharib et al., 2016). Patients were chosen according to the following criteria: (1) cystic nodules or predominantly cystic nodules (>50% fluid components on ultrasound); (2) cytology confirmed benign thyroid nodules with maximum diameter larger than 10 mm in ultrasound; (3) cosmetic problems or pressure symptoms but no dyspnea (vital signs were stable); (4) normal serum thyroid hormones and TSH level. The following exclusion criteria were also used: (1) reluctance to receive USG-LIA; (2) pregnancy; (3) coagulopathy (international normalized ratio > 1.5, platelets < 50,000); (4) lost follow-up. Finally, a total of 107 nodules in 102 patients were enrolled in this study. Thirty of them were male and 72 were female, aged 18–72 years (mean age, 50 ± 13 years).

### Ultrasound Examination

All patients were fully informed of the advantages and disadvantages of surgery, ultrasound guided radiofrequency ablation (USG-RA) and USG-LIA in the treatment of thyroid nodules. Written informed consent was provided by each patient before procedure. Thyroid ultrasound examination and ultrasound-guided fine-needle aspiration cytology were performed before USG-LIA. Both procedures were performed by one of the two radiologists with more than 10, 20 years of ultrasound experiences and more than 5, 10 years of intervention experiences, respectively. Thyroid ultrasound examination was performed by using the Resona 7 ultrasound system (Mindray Medical International, Shenzhen, China) equipped with the L14-5 and L11-3 high frequency linear array probes. Gray-scale ultrasound images of the target thyroid nodule in transversal and longitudinal section were obtained. Ultrasound features of target nodule including size, location, shape, margin, and components were assessed. Volume of nodules was calculated using the following equation: *V* = π abc / 6, where *V* is the volume, *a* is the largest diameter in longitudinal axis, and *b* and *c* are the other two diameters in transversal axis.

### USG-LIA Procedure

Patients were placed in a supine position with mild neck extension. Ultrasound- guided fine-needle aspiration was performed by one radiologist using a 25-gaged needle. The samples from aspirated fluid in purely cystic nodules or solid components in predominantly cystic nodules were sent for cytology. USG-LIA was performed after the benign cytological result was reported. In general, USG-LIA was performed by two radiologists. One radiologist handled the probe with one hand, and a 18–22 gauge needle fitted to a T-junction with another hand. After regional skin sterilization but no anesthesia, the radiologist chose the best puncture path and inserted the needle tip into the target nodule under ultrasound guidance via the trans-isthmic approach. Then, the other radiologist connected a 20-ml plastic syringe with the T-junction and sucked the cystic fluid from the nodule. After the maximum volume of internal fluid was aspirated, lauromacrogol (Lauromacrogol Injection, 10 mL: 100 mg; Tianyu Pharmaceutical Co Ltd., Shanxi, China) was injected into the nodule. For nodules that were difficult to aspirate due to viscous cystic fluid, normal saline was used to lavage the cavity for 2–3 times before lauromacrogol injection. The volume of injected lauromacrogol usually corresponded to about 30–50% of the volume of aspirated fluid. Then, the needle was withdrawn slowly with minimal negative pressure of the syringe, thus preventing lauromacrogol leakage outside the thyroid gland. Volumes of aspirated fluid and injected lauromacrogol were recorded for each nodule. Patients were asked to stay for observation at least 30 min after the procedure. Images of the nodules and the procedures of USG-LIA were stored on both local hard disk and DICOM system.

### Follow-up

Follow-up ultrasound examinations were performed 1st, 3rd, 6th, 12th month and a 6 months interval from 2nd year after the initial procedure. Any complications during follow-up were evaluated. Volume and ultrasound feature of treated nodule were assessed and recorded. Volume reduction rate (VRR) was defined as (initial volume − last volume)/ initial volume. If the volume reduction rate was less than 20% at the 3-month follow-up, repeated USG-LIA was performed again and the corresponding follow-up was also performed. If the reduction rate was less than 50%, and the solid part of the nodule is rich in blood supply, USG-RA or surgery was recommended, and the last volume of these nodules was set as the volume before USG-RA or surgery treatment. The resolution rate was defined as VRR > 50%. VRR < 50% was defined as ineffective, 50–90% was defined as effective, and > 90% was defined as cured.

### Statistical Analysis

Statistical analyses were performed using SPSS version 17.0 (SPSS Inc., Chicago, IL, United States). Wilcoxon signed ranks test was used to compare initial and last volume and maximum diameter of the treated nodule before and after USG-LIA. This test was also used to compare volume changes during follow-up (initial volume vs. 1st month, 1st vs. 3rd month, 3rd vs. 6th month, 6th vs. 12th month, and 12th month vs. last volume). Mann-Whitney U test was used to assess the efficacy between purely cysts and predominantly cystic nodules. The predetermined level of significance was set at a *P* value of 0.05.

## Results

**FIGURE 1:**
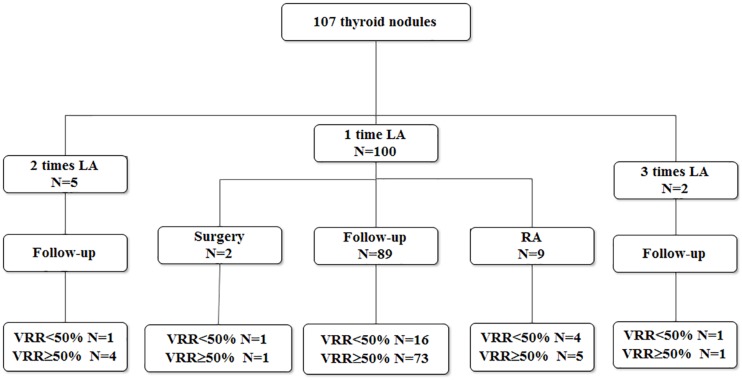
Results of 107 thyroid nodules treated with USG-LIA.

There were 24 purely cystic (22.22%) and 83 predominantly cystic nodules (77.78%). The cytology results of these 107 nodules included 101 thyroid cysts, 5 goiters with cystic change, 1 adenoma with cystic change. One hundred of 107 (93.46%) nodules treated by one session of USG-LIA, 5 (4.67%) underwent 2 sessions of USG-LIA, 2 (1.87%) underwent 3 sessions of USG-LIA (Figure 1). The average volume of aspiration was 15.6 ml, range from 0.4 to160 ml, and mean volume of injected lauromacrogol was 6.37 ml, range from 0.2 to 40 ml. The medium of follow-up period was 10.2 months, range from 1 to 24 months (Table 1).

**TABLE 1:** Baseline characteristics of 107 nodules treated with USG-LIA.

**Characteristics**	**Results**
Mean age, y (SD)	50 (13)
Sex, *n*	
Male	30
Female	72
Component, *n*	
Purely cysts	24
Predominantly cystic	83
Watery/Viscous, *n*	89/18
Volume of aspiration, mean (range)	15.6, (0.4–160)ml
Volume of remained lauromacrogol, mean (range)	6.37, (0.2–40)ml
Follow-up period, mean (range)	10.2, (1–24)month
	

The effectiveness of USG-LIA according to initial volume of 107 nodules was listed in Table 2. The average initial volume and maximum diameter of nodules was 17.27 ± 20.51 ml and 37.92 ± 12.44 mm. The average last volume and maximum diameter of nodules was 5.35 ± 14.68 ml and 21.63 ± 12.39 mm. There was significance of volume and diameter reduction before and after treatment (*P* = 0.000 for both).

**TABLE 2:** Results of 107 nodules treated with USG-LIA.

**Characteristics**	**Mean ± SD**	**Range**	***P* value**
Initial volume	17.27±20.51	0.38–146 ml	0.000
Last volume	5.35±14.68	0–139.95 ml	
Initial diameter (maximum)	37.92±12.44	10.5–88.0 mm	0.000
Last diameter(maximum)	21.63±12.39	0–77.2 mm	

**FIGURE 2:**
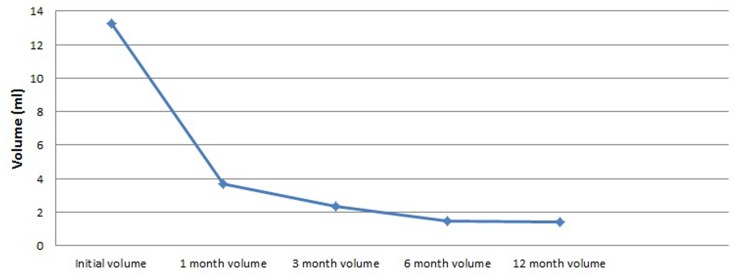
Volume changes of 43 thyroid nodules after USG-LIA with more than 12 months follow-up.

We analyzed volume changes of 43 nodules that follow-up at least 12 months and listed them in Table 3 and Figure 2. After USG-LIA, the average volume reduced from 13.24 ± 15.46 ml (initial volume) to 3.68 ± 6.47 ml (1st month), 2.33 ± 4.84 (3rd month) and 1.46 ± 2.84 (6th month). By the time of terminal follow-up, the average volume reduced to 1.39 ± 2.84 ml. Within 6 months after treatment, the volume of the target nodule at each follow-up was smaller than the previous one (*P* < 0.001 for all). However, there was no significant difference of volume change between 6th month and 12th month.

**TABLE 3:** Volume changes of 43 nodules with more than 12 months follow-up period.

**Characteristics**	**Mean ± SD(ml)**	**Range**
Initial volume	13.24±15.46	0.38–88.26 ml
1 month volume	3.68±6.47	0.19–37.38 ml
3 month volume	2.33±4.84	0.1–27.23 ml
6 month volume	1.46±2.84	0.03–14.14 ml
12 month volume	1.40±2.84	0.04–14.14 ml

**FIGURE 3:**
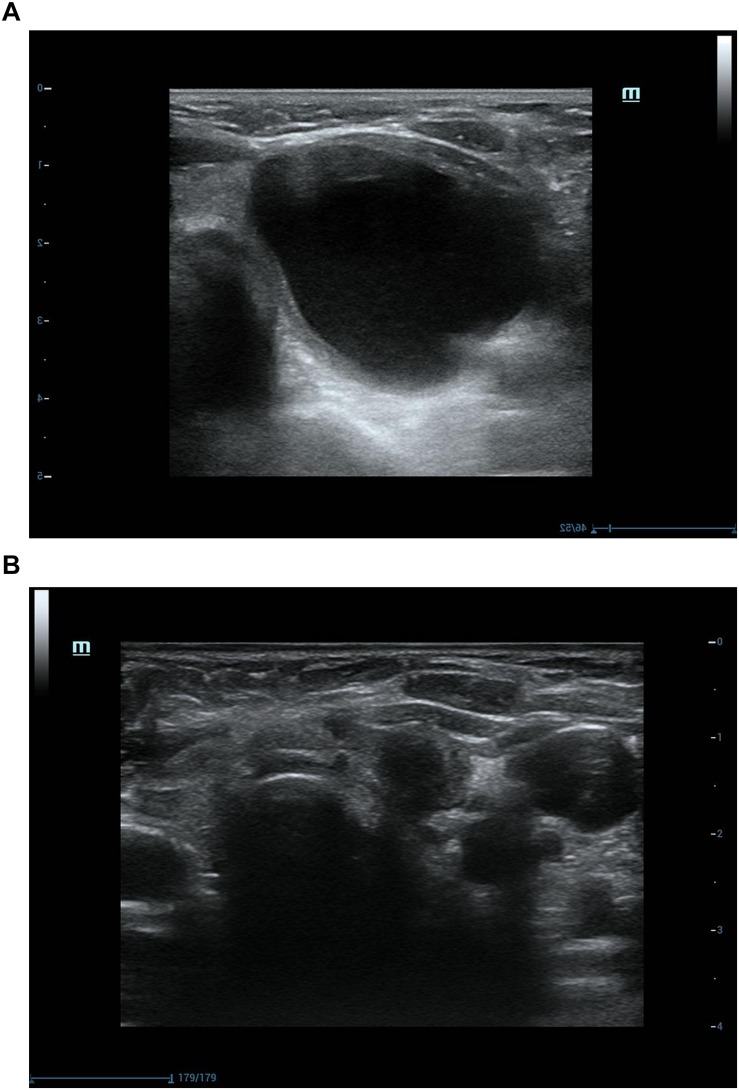
Ultrasonograpic image of a 70 years old female before and after treatment.**(A)** A purely cystic nodule of sonography before USG-LIA.**(B)** 18 months after USG-LIA.

**FIGURE 4:**
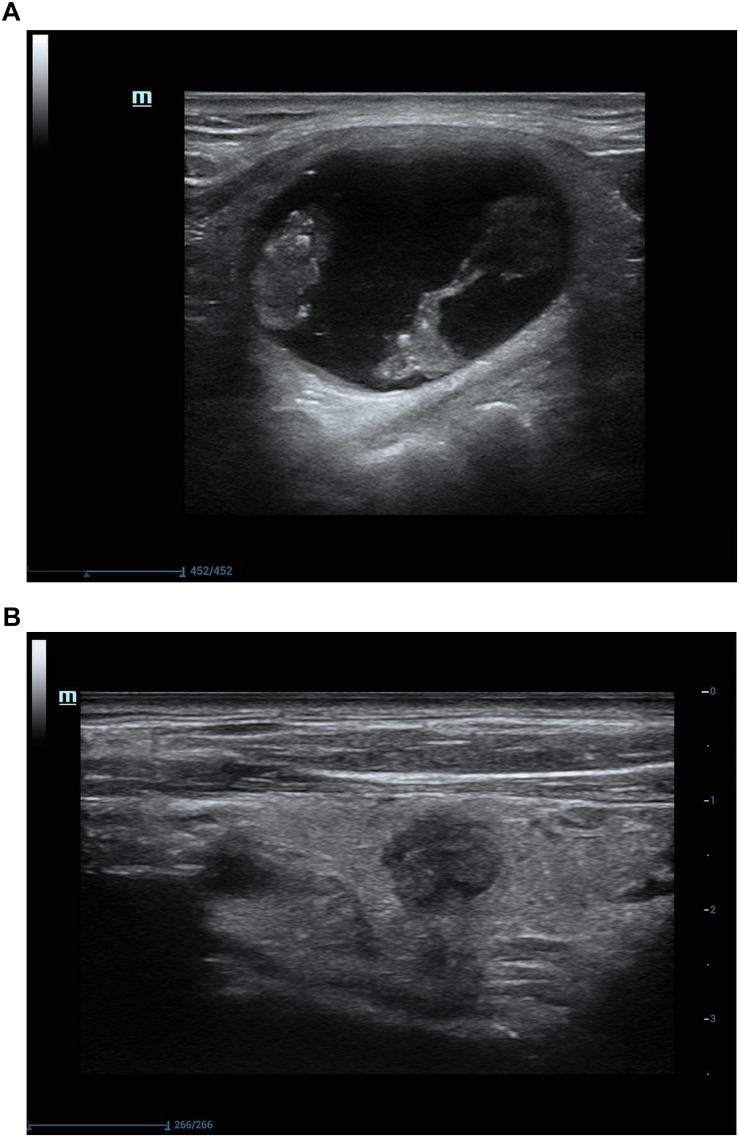
Ultrasonograpic image of a 62 years old male before and after treatment.**(A)** A predominantly cystic nodule before USG-LIA.**(B)** 12 months after USG-LIA.

Among 107 nodules treated by USG-LIA, 85 nodules (79.44%) had volume reduced rate > 50% and no recurrence by the end of the study. The overall resolution rate (VRR > 50%) was 91.67% in purely cysts and 75.90% in predominantly cystic nodules. The ineffective rate (VRR < 50%), effective rate (VRR 50–90%) and cured rate (VRR > 90%) were 8.33, 37.5, 54.17% in purely cysts and 24.10, 40.96, and 34.94% in predominantly cystic nodules, respectively (Table 4 and Figures 3, 4). There was significant difference in efficacy rate between purely cystic nodules and predominantly cystic nodules (*P* = 0.047).

**TABLE 4:** Efficacy of USG-LIA treatment in purely cysts and Predominantly cystic nodules.

**Nodule Characteristics**	**Volume reduction**			**Total**
	**<50%**	**50–90%**	**>90%**	
Purely cysts	2	9	13	24
	(8.33%)	(37.5%)	(54.17%)	(100%)
Predominantly cysts	20	34	29	83
	(24.10%)	(40.96%)	(34.94%)	(100%)
Total	22	43	42	107
	(20.56%)	(40.19%)	(39.25%)	(100%)

Twenty-two out of 107 nodules had a VRR < 50%. Among them, 20 were predominantly cystic nodules and only 2 of them were purely cysts. Of the 100 nodules that performed 1 session of USG-LIA, 20 of them had a VRR < 50%. Of the 5 that performed 2 sessions, 1 had a VRR < 50%. Of the 2 that performed 3 sessions, 1 had a VRR < 50%. The overall resolution rate (VRR > 50%) after 1 session, 2 sessions and 3 sessions of USG-LIA was 74.77% (80/107), 78.50% (84/107), 79.44% (85/107). Of the 7 nodules that performed 2–3 sessions of USG-LIA, 5 were predominantly cystic nodules.

### Complications

Mild or moderate fever (37.5–39°C) with mild neck pain occurred in 5.9% patients (6/102) after treatment of USG-LIA within 3 days. All symptoms disappeared and patients recovered within 1 week after symptomatic treatment. No severe complications occurred in this study.

## Discussion

Cystic nodules are almost impossible to be malignant, and partially cystic nodules without any suspicious ultrasound features have a malignancy rate of < 3% (Haugen et al., 2016). It is generally considered that USG-PEI is a safe and effective therapy for benign purely cystic and predominantly cystic thyroid nodules (Gharib et al., 2016; Haugen et al., 2016). Lauromacrogol, a new sclerosing agent different from absolute alcohol was used in this study. Lauromacrogol injection is an effective sclerosing agent consisting of polyoxyethylene lauryl ether [molecular formula:C12H25(OCH2CH2)nOH (*n* = 9), formula weight:582.8], with accessories of alcohol and water. Lauromacrogol injection has been used in hemorrage of esophago gastric varices and varix of lower limb for a decade. It was believed that this sclerosing agent was safe and effective in treating heptatic cysts and renal cysts (Dell’Atti, 2015; Xue and Geng, 2015; Wijnands et al., 2018). Animal model experiments showed that lauromacrogol may destroy thyroid endothelial cells on the capsule wall of the cysts, leading to aseptic inflammation and increasing fibrosis (Idiz et al., 2016).

Our study found that there was a significant difference in efficacy rate of USG-LIA between purely cystic nodules and predominantly cystic nodules (*P* = 0.047). Results revealed that USG-LIA had excellent performance in the treatment of purely cysts, of which over 90% had an effective volume shrinkage (VRR > 50%), and more than half had been cured (VRR > 90%). As a contrast, in predominantly cystic nodules, 75.9% of the nodules had an effective volume shrinkage, and 34.9% had been cured. Kim reported that USG-PEI had superior effect in the treatment of purely cysts than predominantly cystic nodules (therapeutic success rate: 90.3 vs. 82.2%) (Kim et al., 2012). In this study, we obtained similar results (91.67 vs. 75.90%).

It was reported that USG-PEI had a 75–85% success rate in treating cystic nodules and 17.9–21% of USG-PEI patients had side effect such as mild pain, fever, blush, and dizziness (Bennedbaek and Hegedus, 2003; Valcavi and Frasoldati, 2004; Mauz et al., 2005; Suh et al., 2015). Comparing these with USG-PEI, our results demonstrated that USG-LIA had equivalent effects but fewer complications. Only 5.9% (6/102) of patients complained of mild-to-moderate fever with mild neck pain.

We found that the volume of the treated nodules gradually shrank within 6 months (*P* < 0.001 for all). However, 6 months after USG-LIA, the average volume of treated nodules reduced less obviously. Therefore, it seems reasonable that the next management plan of each nodule be decided in the 6th month follow-up. In this study, seven patients had more than two sessions of USG-LIA treatment and finally five of them had VRR > 50%. Therefore, repeated USG-LIA procedures should be performed in some intractable nodules.

This study was characterized by some limitations. First, our work was a single-center prospective study. Second, part of the patients (20/102) had a short follow-up period (1 months), which might cause under-estimation of effectiveness. Therefore, further studies are needed to verify our results.

In conclusion, our study demonstrates that USG-LIA is an effective and safe treatment modality in both purely cystic and predominantly cystic thyroid nodules, and can be used as an alternative to surgery.

## Author Contributions

JZ, WZ designed the study. ZL, TL acquired the data. YD contributed manuscript and analysis. JZ revised the manuscript.

## Conflict of Interest Statement

The authors declare that the research was conducted in the absence of any commercial or financial relationships that could be construed as a potential conflict of interest.
